# The disruptive effects of COPD exacerbation-associated factors on epithelial repair responses

**DOI:** 10.3389/fimmu.2024.1346491

**Published:** 2024-06-07

**Authors:** Rosa K. Kortekaas, Kerstin E. Geillinger-Kästle, Rocío Fuentes-Mateos, Roël van Orsoy, Nakaa Al-Alyan, Janette K. Burgess, Reinoud Gosens

**Affiliations:** ^1^ Department of Molecular Pharmacology, University of Groningen, Groningen, Netherlands; ^2^ Groningen Research Institute for Asthma and COPD (GRIAC), University Medical Center Groningen, University of Groningen, Groningen, Netherlands; ^3^ Department of Immunology and Respiratory Diseases Research, Boehringer Ingelheim Pharma GmbH & Co. KG, Biberach an der Riss, Germany; ^4^ Department of Pathology and Medical Biology, University Medical Center Groningen, University of Groningen, Groningen, Netherlands

**Keywords:** COPD, exacerbation, inflammation, spheroids, regeneration, stem cell niche, epithelial cells

## Abstract

**Introduction:**

Exacerbations of chronic obstructive pulmonary disease (COPD) increase mortality risk and can lead to accelerated loss of lung function. The increased inflammatory response during exacerbations contributes to worsening of airflow limitation, but whether it also impacts epithelial repair is unclear. Therefore, we studied the effect of the soluble factor micro-environment during COPD exacerbations on epithelial repair using an exacerbation cocktail (EC), composed of four factors that are increased in COPD lungs during exacerbations (IL-1β, IL-6, IL-8, TNF-α).

**Methods:**

Mouse organoids (primary CD31-CD45-Epcam+ cells co-cultured with CCL206 fibroblasts) were used to study epithelial progenitor behavior. Mature epithelial cell responses were evaluated using mouse precision cut lung slices (PCLS). The expression of epithelial supportive factors was assessed in CCL206 fibroblasts and primary human lung fibroblasts.

**Results:**

EC exposure increased the number and size of organoids formed, and upregulated *Lamp3*, *Muc5ac* and *Muc5b* expression in day 14 organoids. In PCLS, EC imparted no effect on epithelial marker expression. Pre-treatment of CCL206 fibroblasts with EC was sufficient to increase organoid formation. Additionally, the expression of *Il33*, *Tgfa* and *Areg* was increased in CCL206 fibroblasts from EC treated organoids, but these factors individually did not affect organoid formation or size. However, TGF-α downregulated *Foxj1* expression and upregulated *Aqp5* expression in day 14 organoids.

**Conclusions:**

EC exposure stimulates organoid formation and growth, but it alters epithelial differentiation. EC changes the epithelial progenitor support function of fibroblasts which contributes to observed effects on epithelial progenitors.

## Introduction

Chronic obstructive pulmonary disease (COPD) is a heterogeneous lung disease with progressive airflow limitation (1). Exacerbations of COPD are associated with an acute worsening of symptoms (2), such as coughing, increased sputum production and dyspnea (1). COPD patients with more severe airflow limitation and prior exacerbations are at a higher risk of developing an exacerbation, while significant air pollution or viral and bacterial infections may trigger an exacerbation (2). Exacerbations are significant events in the trajectory of COPD patients since they often require hospitalization and are associated with worsening outcomes. In particular, severe exacerbations are associated with increased mortality (3) and COPD patients who experience frequent exacerbations have a greater rate of decline in forced expiratory volume in 1 second (FEV1) (4). Exacerbations are even estimated to account for as much as 25% of the lung function decline in patients with COPD (5). Moreover, while the immediate loss of lung function that occurs during an exacerbation improves over time, it often does not return to the pre-exacerbation levels (6). Specifically, in 25% of exacerbators lung function is not restored to pre-exacerbation levels after 35 days. In 7% of COPD patients experiencing an exacerbation, lung function has still not recovered to baseline after 91 days (7).

During a COPD exacerbation, higher concentrations of inflammatory cytokines and factors are found in sputum (8, 9). The increased inflammatory response during an exacerbation contributes to the development of airway oedema, mucus hypersecretion and worsening of airflow limitation (10). Whether the increased inflammation following an exacerbation also affects other processes in the lung such as epithelial repair has not yet been explored. Endogenous epithelial progenitor cells, which are responsible for epithelial repair responses, are dependent on cues from their micro-environment, including soluble factors and their communication with surrounding cells (11). Fibroblasts in particular are highly important in supporting epithelial progenitor cell function through the secretion of paracrine factors including Wnt ligands and fibroblast growth factors (FGFs) (11, 12). We hypothesize that epithelial progenitors develop an altered response to a pro-inflammatory soluble factor milieu during exacerbations, and that an altered epithelial progenitor support function of fibroblasts contributes to this process.

Here, we mimicked the soluble factor micro-environment during a COPD exacerbation using an exacerbation cocktail (EC), composed of factors which were found to be increased during an exacerbation compared to stable COPD. The cocktail consisted of interleukin-1 beta (IL-1β), IL-6, IL-8 and tumor necrosis factor alpha (TNF-α), in concentrations found in the sputum of COPD patients during an exacerbation (9, 13). Using EC we aimed to investigate whether the changed soluble factor micro-environment during a COPD exacerbation influences epithelial repair responses, by using mouse organoids and precision cut lung slices (PCLS). We also used pre-exposed fibroblasts in organoid cultures to ask if EC alters the progenitor support function of fibroblasts, thereby affecting epithelial repair. Finally, we selected three soluble factors (IL-33, amphiregulin and transforming growth factor α (TGF-α)) that were significantly altered in the fibroblast by EC exposure, and studied their impact on epithelial repair in more detail.

## Methods

### Animal handling

Specific pathogen-free wild type C57BL/6J mice (>8 weeks old, male and female) were housed in an environment with controlled humidity at 24 ± 1°C under a 12 hour light/dark cycle. Water and food were given ad libitum. All animal procedures were approved by the local Animal Care and Use committee of the University of Groningen and performed according to the national guidelines under approval number AVD1050020209205.

### Ethics human samples

The procedure for obtaining human lung tissue was in accordance to the Research Code of the University Medical Center Groningen (UMCG), as stated on https://umcgresearch.org/w/research-code-umcg as well as national ethical and professional guidelines Code of Conduct for Health Research (https://www.coreon.org/wp-content/uploads/2023/06/Code-of-Conduct-for-Health-Research-2022.pdf). The use of left-over lung tissue in this study was not subject to Medical Research Human Subjects Act in the Netherlands, as confirmed by a statement of the Medical Ethical Committee of the University Medical Center Groningen and therefore exempt from consent according to national laws (Dutch laws: Medical Treatment Agreement Act (WGBO) art 458/GDPR art 9/UAVG art 24). All donor material and clinical information were deidentified prior to experimental procedures, blinding any identifiable information to the investigators. Human lung tissue was obtained from excess lung tissue surplus to clinical care requirements derived from lung resection and lung transplant procedures. Histologically normal lung tissue from tumor resection surgery (non-COPD) was obtained from an area as far away as possible from the tumor and all tissues were examined by an experienced pathologist to exclude the presence of abnormalities.

### Fibroblast cultures

The mouse lung fibroblast cell line CCL206 (CCL-206; ATCC, Wesel, Germany, RRID: CVCL_0437) was cultured in CCL206 medium; DMEM (Lonza, Basel, Switzerland, #42430)/Ham’s F12 (Gibco, #21765) (1:1) supplemented with 10% (v/v) fetal bovine serum (FBS, PAA Laboratories, Pasching, Austria), 2 mM L-glutamine (Life Technologies, Carlsbad, CA, USA, #35050–061), 100 U/ml penicillin/streptomycin (Gibco, Waltham, MA, USA, #15070-063) and 1% amphotericin B (Gibco, #15290-026). Primary human lung fibroblasts were obtained from non-COPD lung material using the crawl out method, as previously described (14). They were maintained in primary fibroblast medium (low glucose DMEM (Biowest, Nuaillé, France) with 10% FBS, 100 U/ml penicillin/streptomycin and 1% glutamax (Gibco, #35050-061)), and used at passage 5 for all experiments. **Table 1** summarizes the characteristics of the fibroblast donors of all experiments. All cells were maintained at 37°C with 5% CO_2_ and confirmed to be mycoplasma negative before use.

**Table 1:** Donor characteristics of primary human non-COPD fibroblasts used in gene expression studies.

Age Median (range)	Sex (M/F)	Smoking status Never/current/ex	Pack years Median (range)	FEV1% of predicted Median (range)	FVC % of predicted Median (range)	FEV1/FVC Median (range)
65 (60-68)	6/0	0/0/6	42.5 (40-70) 2 unknown	83.4 (61.4 - 90.7)	83 (58.4 - 89.5) 1 unknown	0.76 (0.70-0.79)

### Mouse Epcam+ isolation for organoid culture

Epcam+ cells were isolated as described previously (15). Briefly, the lungs of adult C57BL/6J mice were instilled with dispase (BD Biosciences, Oxford, UK, #354235) and digested for 45 minutes at 37°C, after which they were further digested with 220 units/ml DNAse1 (Applichem, Darmstadt, Germany #A3778) in DMEM, and filtered through cell strainers (Corning Incorporated, Corning, NY, USA, #431752). The resulting cell suspension was depleted of CD31+ and CD45+ cells using CD31 (Miltenyi, #130-097-418, RRID: AB_2814657) and CD45 (Miltenyi Biotec, Teterow, Germany #130-052-301, RRID: AB_2877061) microbeads in combination with LS columns (Miltenyi #130-091-051). Finally, the suspension was enriched for epithelial cells using CD326 (Epcam) microbeads (Miltenyi #130-105-958).

### AT2 cell enrichment for organoid culture

After digesting the lungs of adult mice and depleting the cell suspension of CD31+ and CD45+ cells (as described above), the cell suspension was divided into three fractions. 1) The first fraction was incubated with CD326 (Epcam) microbeads to obtain epithelial cells as described above (CD31-CD45-Epcam+ fraction). 2) The second fraction was enriched for alveolar type 2 cells (AT2) cells using MHCII-PE antibody (BD Biosciences, #557000) and anti-PE microbeads (Miltenyi, #130-048-801) (CD31-CD45-MHCII+ fraction). 3) The third fraction was also enriched for AT2 cells, with a different approach. This fraction was first incubated with CD326 (Epcam) microbeads and subsequently enriched using MHCII-PE antibody and anti-PE microbeads (CD31-CD45-Epcam+MHCII+ fraction).

### Organoid culture

The organoid culture was based on published protocols (15). Confluent CCL206 fibroblasts were treated with 10 μg/ml mitomycin C (Sigma-Aldrich, St. Louis, MO, USA, #M4287) for 2 hours in a culture flask to inhibit their proliferation. They were then allowed to recover in normal medium for at least 1 hour, after which they were harvested. Subsequently, 10,000 CCL206 fibroblasts were mixed with 10,000 freshly isolated mouse CD31-CD45-Epcam+ cells, CD31-CD45-MHCII+ cells or CD31-CD45-Epcam+MHCII+ cells. The cell mixtures were seeded into inserts for 24-well plates (Thermo Fisher Scientific, Waltham, USA #10421761) in growth-factor reduced Matrigel (Fisher Scientific, Landsmeer, The Netherlands #11523550) mixed 1:1 with CCL206 medium, and cultured for 14 days with DMEM/Ham’s F12 supplemented with 5% FBS, 100 U/ml penicillin/streptomycin, 1% amphotericin B, 2 mM L-glutamine, insulin-transferrin-selenium (Gibco #15290018), bovine pituitary extract (30 μg/ml, Sigma-Aldrich, #P1476), and recombinant mouse EGF (0.025μg/ml, Sigma-Aldrich, #SRP3196). Y-27632 (10 μM, Tocris Bioscience, Oxford, UK, #1254) was also added for the first 48 hours of culture. Medium was replaced every 2-3 days. Organoid cultures were treated with exacerbation cocktail (see **Table 2**) or the compounds listed in **Table 3** during 14 days of culture. Organoid number and size were quantified on day 14, after which organoids were either fixed with acetone/methanol at -20°C for 15 minutes and stored for subsequent immunofluorescent staining or collected for RNA isolation.

**Table 2:** Composition of the exacerbation cocktail (prepared as a 100x stock in 0.1% BSA in PBS).

Component	Final concentration	Product information
IL-1β	300 pg/ml	IL038, Sigma-Aldrich (St. Louis, MO, USA)
IL-6	300 pg/ml	200-06, Peprotech (Cranbury, NJ, USA)
IL-8 (human) Or KC (mouse)*	20 ng/ml 20 ng/ml	200-08M, Peprotech 250-11, Peprotech
TNFα	200 pg/ml	T6674, Sigma-Aldrich

*Species-matched exacerbation cocktail was used throughout the studies, containing IL-8 for human cells or KC for mouse cells

**Table 3:** List of treatments used on mouse organoids.

	Final concentration	Product information
Amphiregulin	10 ng/ml and 50 ng/ml	315-36, Peprotech
TGF-α	10 ng/ml and 50 ng/ml	100-16A, Peprotech
Gefitinib (Iressa^®^)	1 μM and 10 μM	3000, R&D systems (Minneapolis, MN, USA)
IL-33	10 ng/ml	200-33, Peprotech
IL-33R antibody	1.5 μg/ml	MAB523, R&D systems

### Cytospins

50.000 freshly isolated CD31-CD45-Epcam+ cells, CD31-CD45-MHCII+ cells and CD31-CD45-Epcam+MHCII+ cells were spun down onto six 3% (w/v) bovine serum albumin (BSA, Capricorn Scientific, Ebsdorfergrund, Germany) pre-coated microscope slides for 5 min at 550 rpm. Cytospins were air-dried for 30 min and subsequently stored at -20 °C. Samples were permeabilized with 0.1% Triton X in PBS for 10 min, and subsequently blocked for 1 hour with 5% BSA, 2% donkey serum (Jackson Immunoresearch, West Grove, PA, USA, #017-000-121, RRID: AB_2337258), 0.1% Triton X in PBS. Next, samples were incubated overnight at °4 C with rabbit anti-Prosurfactant Protein C (pro-SPC) (MiliporeSigma, Burlington, MA, USA, #AB3786, RRID: AB_91588) diluted 1:1000 in 2% BSA, 2% donkey serum, 0.1% Triton in PBS. They were then washed three times with PBS, and subsequently incubated for 1 hour with the secondary antibody donkey anti-rabbit Alexa fluor 488 (Thermo Fisher Scientific #A21206, RRID : AB_2535792) diluted 1:500. After washing the samples three times with PBS, cytospins were covered with mounting medium containing 4’,6-diamidino-2-phenylindole (DAPI) (Abcam, #104139) and visualized with an Eclipse Ti2-E microscope (Nikon, Tokyo, Japan) at magnification 20x. Representative images were magnified from original 20x images, but were otherwise not altered. Mean Fluorescence Intensity (MFI) was analyzed using ImageJ software (16) by defining the region of interest of each cell and measuring the MFI per cell for pro-SPC staining.

### Immunofluoresence

Mouse organoids were stained as previously described (15). Briefly, fixed organoids were blocked inside the insert with 5% BSA in PBS for 2 hours, and then incubated with primary antibodies rabbit anti-pro-SPC (MilliporeSigma, #AB3786) and mouse anti-Acetylated α Tubulin (ACT) (Santa Cruz Biotechnology, Dallas, TX, USA, #sc-23950, RRID: AB_628409) diluted 1:200 in 0.1% BSA 0.1% Triton X in PBS at 4°C overnight. Subsequently, secondary antibodies donkey anti-rabbit Alexa fluor 488 (Thermo Fisher Scientific #A21206, RRID: AB_2535792) and donkey anti-mouse Alexa fluor 568 (Thermo Fisher Scientific #A10037, RRID: AB_2534013) diluted 1:200 in 0.1% BSA 0.1% Triton X in PBS were added for 2 hours. Fixed organoid cultures were then excised from the insert and mounted on glass slides with mounting medium containing DAPI(Abcam). Organoid staining was quantified using a blinded approach on a DM4000B fluorescence microscope (Leica Camera, Wetzlar, Germany).

### Precision cut lung slices (PCLS)

PCLS were obtained as previously described (17). Briefly, the lungs of adult C57BL/6J mice were inflated with 1.5% low-melting point agarose (Gerbu Biotechnik GmbH, Wieblingen, Germany), separated into lobes, and cut into PCLS of 250 μm thickness using a vibrating microtome (VT1000 S Vibrating blade microtome, Leica). Slices were placed into 12-well plates and treated with vehicle control or EC for 48 hours. They were then stored at -80°C until further processing for RNA isolation, which was performed using Maxwell simplyRNA tissue kit (Promega, Madison, WI, USA, #AS1280) according to manufacturer’s instructions.

### Organoid re-sorting

300,000 proliferation-inactivated CCL206 fibroblasts were mixed with 300,000 freshly isolated mouse Epcam+ cells and seeded into 6-well plates in 500 μl growth factor reduced Matrigel mixed with 500 μl CCL206 medium. They were cultured for 72 hours with mouse organoid medium supplemented with Y-27632 and in the presence of vehicle control or EC, after which the Matrigel was disrupted by 45 minutes incubation with dispase (1:1). Organoids were brought into a single cell suspension by incubating with trypsin for 5 minutes. Cells were then sorted back into Epcam+ cells and Epcam- cells (CCL206 fibroblasts) using CD326 microbeads and LS columns, and both populations were subjected to RNA isolation using the NucleoSpin^®^ RNA kit (Macherey-Nagel, Düren, Germany, #740955) according to manufacturer’s instructions. Finally, both populations were bulk RNA-sequenced separately.

### RNA sequencing

RNA concentrations were initially determined on a Nanodrop ND-1000 spectrophotometer, and quantified in detail by a Bioanalyzer and Fragment analyzer system (Agilent). Genomescan (the Netherlands) used the NEBNext Ultra II Directional RNA Library Prep Kit for Illumina (New England Biolabs, Ipswich, MA, USA #E7760S/L) to process the samples and performed bulk RNA-sequencing using an Illumina NovaSeq 6000 sequencer. Data quality control, adapter trimming, alignment of short reads and feature counting were included in the procedure. Ribosomal (and globin) content was determined to check the library preparation. Samples were checked for possible sample and barcode contamination and various standard quality metrics of the raw data set were determined using FstQC v0.34 and FastQA. The reads were trimmed for adapter sequences using Trimmomatic v0.30 and then aligned with the mouse reference GRCm38 (patch 6). Gene duplicates and genes with an average raw count <1 were removed. Paired-sample analysis using the DESeq2 package 1.34.0 in R 4.2.2 was performed to determine differentially expressed genes (DEGs), which were regarded as significant when padj<0.05. Pathway enrichment analysis was done with all DEGs using the fgsea package 1.20.0 in R. Pathways were considered significant when padj<0.05. Volcano plots were generated using the R package EnhancedVolcano 1.14.0 with cut-offs padj<0.05 and logFold>1. ComplexHeatmap version 2.12.1 was used to create heatmaps with normalized counts obtained from DESeq2 analysis as input. Normalized counts obtained from DESeq2 analysis were also used to report normalized count values. Epithelial signature scores were determined using the marker genes associated with specific epithelial subtypes (from the human lung cell atlas (18)). These gene sets were used as pathway definitions in order to run pathway enrichment analysis with fgsea in R. The complete dataset is available via the Geo Dataset GSE245259.

### Gene expression studies fibroblasts

Primary human fibroblasts of non-COPD donors (**Table 1**) were seeded into 6-well plates in primary fibroblast medium at a density of 300,000 cells per well. They were then quiesced for 24 hours in low glucose DMEM with 0.1% BSA and antibiotics, and then exposed to EC for 24 hours, after which they were lysed in TRIzol (Invitrogen, Waltham, MA, USA, #15596018). RNA was isolated with TRIzol according to manufacturer’s instructions.

### RT-qPCR

RNA concentrations were quantified with a Nanodrop ND-1000 spectrophotometer. Equal mRNA concentrations were reverse transcribed into cDNA. A 7900HT Fast Real-Time PCR System (Applied Biosystems, Waltham, MA, USA) was used for Real-Time qPCR, with denaturation at 94°C for 30 seconds, annealing at 60°C for 30 seconds and extension at 72°C for 30 seconds for 40 cycles followed by 5 minutes at 72°C, using SYBR green (Roche Applied Science, Mannheim, Germany) as the DNA binding dye. The 2^-ΔΔCT^ method was used to determine fold changes. Gene expression was normalized to housekeeping genes *B2M*, *SDHA* and *HMBS* for human samples and *Rpl13a, B2m* and *Actb* were used as housekeeping genes for mouse samples. The complete lists of human and mouse primer sequences (Biolegio, Nijmegen, the Netherlands) are summarized in **Supplementary Tables 1**, **2** (**Supplementary Material**) respectively.

### Data analysis

GraphPad Prism 8 was used for statistical testing of all data except RNA sequencing data (see above). The number of biological replicates is described by N. Data are presented with mean and standard deviation unless described otherwise. Normalized data were log transformed before statistical evaluation. All data were checked for normality using the Shapiro-Wilk test. For parametric data, a paired two-tailed t-test, paired one-way ANOVA or two-way ANOVA was used, whereas for nonparametric data, a Wilcoxon or Kruskal-Wallis test was used, where appropriate. In cases where there were missing values an unpaired statistical approach was used. Differences were considered significant when p<0.05.

## Results

### The exacerbation cocktail promotes organoid formation and growth, and changes epithelial differentiation dynamics

**Figure 1:**
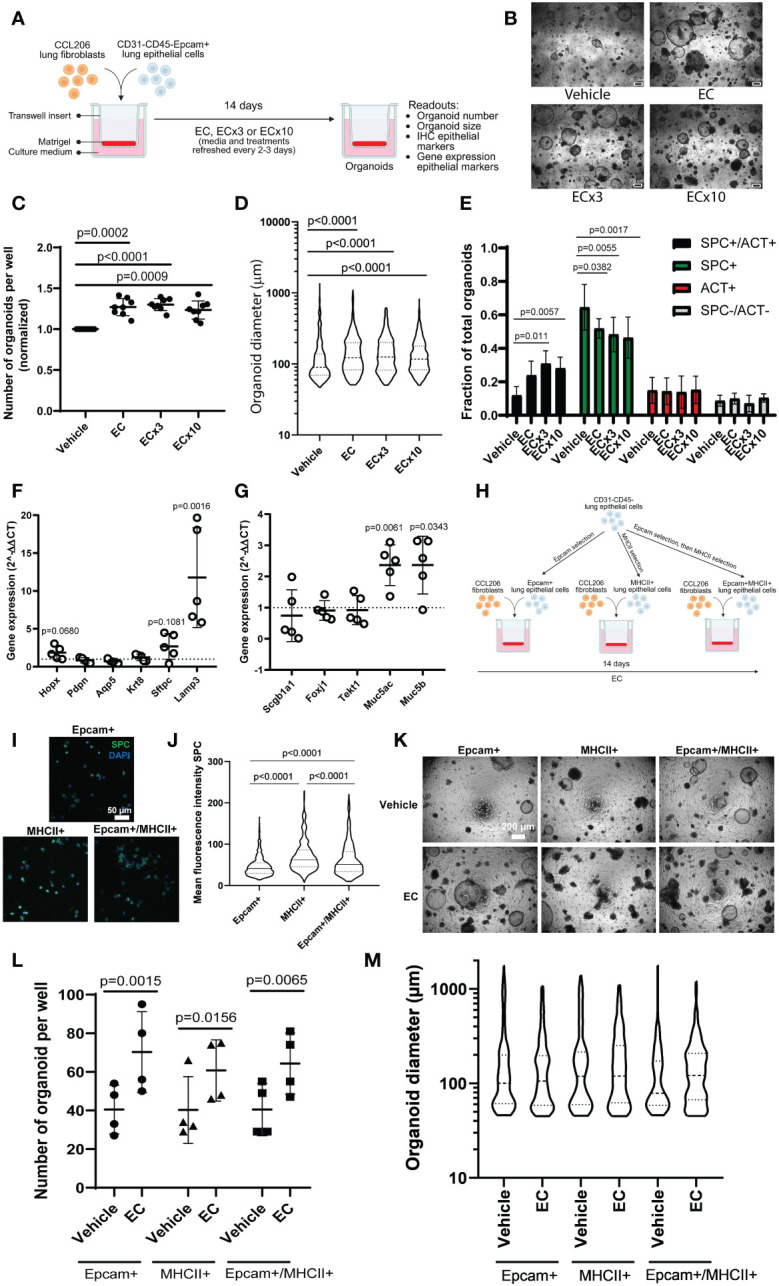
Mouse organoid number and size are increased in response to exacerbation cocktail, associated with altered epithelial differentiation. **(A)** Schematic overview of experimental set-up. CCL206 fibroblasts were mixed with primary CD31-CD45-Epcam+ mouse lung epithelial cells and seeded into Matrigel. Cultures were continuously treated with exacerbation cocktail (EC), ECx3 or ECx10 for 14 days, after which resulting organoid formation was studied (created using BioRender.com). **(B)** Representative brightfield images of day 14 organoids treated with vehicle control and increasing concentrations of EC. Scale bar = 200 μm. **(C)** Number of mouse organoids formed by primary mouse epithelial (Epcam+) cells co-cultured with CCL206 fibroblasts after EC, ECx3 and ECx10 exposure on day 14. Values are normalized to the vehicle control (paired one-way ANOVA on log-transformed data, N=8). **(D)** The effect of EC, ECx3 and ECx10 on mouse organoid size (median is shown, Kruskal-Wallis test, N=8). **(E)** Quantification of immunohistochemistry for pro-SPC and ACT (two-way ANOVA, N=8). **(F)** The effect of EC exposure on the expression of alveolar marker genes *Hopx, Pdpn, Aqp5, Krt8, Sftpc* and *Lamp3* measured by qPCR in day 14 organoids. Values are displayed as 2^-ΔΔCT^ values which were normalized to the vehicle control. Vehicle control value (=1) is represented by dotted line (paired t-test on log-transformed ΔCT values, N=6). **(G)** The expression of proximal epithelial marker genes *Scgb1a1, Foxj1, Tekt1, Muc5ac* and *Muc5b* in day 14 organoids after continuous exposure to EC. Data are presented as 2^-ΔΔCT^ values, normalized to the vehicle control, and vehicle control values (=1) are illustrated by the dotted line (paired t-test on log-transformed ΔCT values, N=6). **(H)** Schematic overview of alveolar type 2 (AT2) cell enrichment approach. Mouse lung cells were depleted of CD31 and CD45 cells, after which the cell suspension was split into three populations. Epithelial cells were enriched by Epcam selection (Epcam+), whereas AT2 cells were enriched by MHCII selection (MHCII+) or by Epcam selection followed by MHCII selection (Epcam+MHCII+). Organoid formation by all three populations was studied (created using BioRender.com). **(I)** Representative images of cytospins from the Epcam+, MHCII+ and Epcam+MHCII+ populations stained with pro-SPC. Scale bar=50 µm. **(J)** Mean fluorescence intensity (MFI) of pro-SPC staining of cytospins (median is shown, Kruskal-Wallis test, N=3. A total of 500-600 cells were assessed per condition). **(K)** Representative brightfield images of organoids formed on day 14 by cells from the three isolation methods in response to EC. Scale bar=200 µm. **(L)** Number of organoids formed on day 14 by Epcam+, MHCII+ or Epcam+MHCII+ cells in response to EC exposure (two-way ANOVA, N=4). **(M)** Organoid diameter measured on day 14 (median is shown, Mann-Whitney test to compare EC to respective vehicle control (Bonferroni-corrected α=0.0167), N=4).

To investigate the effect of the soluble factor milieu during a COPD exacerbation on epithelial growth and differentiation, we first employed the mouse organoid model, which can be used to assess effects on growth of epithelial progenitors into differentiated epithelial cells over the course of 14 days (**Figure 1A**). A concentration response to EC was examined, in which EC approximated the concentrations of factors found in sputum during an COPD exacerbation (9, 13), with two higher concentrations of the cocktail also being included (ECx3 and ECx10) to account for possible dilution of the measured factor concentrations during sample collection. We found that the number and size of the organoids formed by Epcam+ epithelial cells were significantly increased by all EC concentrations tested (**Figures 1B–D**). Quantitative immunohistochemistry for pro-SPC and acetylated tubulin (ACT) revealed that ECx3 and ECx10 significantly increased the fraction of organoids that were pro-SPC+/ACT+ on day 14 (p=0.0011 and p=0.0057 respectively), which was at the expense of pro-SPC+/ACT- organoids (**Figure 1E**). In addition, using qPCR we found that EC significantly upregulated the expression of alveolar type 2 (AT2) cell marker *Lamp3* (p=0.0016) and mucin genes *Muc5ac* (p=0.0061) and *Muc5b* (p=0.0343) in mouse organoids on day 14. The expression of *Sftpc* (marking AT2 cells), as well as markers associated with ciliated cells *(Foxj1, Tekt1)*, club cells *(Scgb1a1)* and AT1 cells *(Hopx, Pdpn, Aqp5)* were not significantly affected by the presence of EC, although *Sftpc* and *Hopx* expression tended to increase (p=0.1081 and p=0.0680 respectively) (**Figures 1F, G**). As the lowest concentration of EC induced a robust response in mouse organoids, we continued our studies with this concentration of EC only. We then aimed to validate the impact of EC on the growth and differentiation of AT2 cells using the organoid model. To do so, we further enriched the starting population for AT2 cells using major histocompatibility complex class II (MHCII) selection (MHCII+) or Epcam followed by MHCII selection (Epcam+MHCII+), and studied the organoid formation of these cell populations compared to the Epcam+ population (**Figure 1H**). Pro-SPC staining of cytospins from these populations revealed that freshly isolated MHCII+ cells and Epcam+MHCII+ cells had a significantly higher expression of pro-SPC compared to Epcam+ cells, indicating further AT2 cell enrichment (**Figures 1I, J**). EC exposure led to a significant increase in the number of organoids formed by all three cell populations to a similar extent (**Figures 1K, L**), and similarly affected organoid size in all three preparations (**Figure 1M**).

Next, we exposed mouse precision cut lung slices (PCLS) to EC for 48 hours to investigate the effect of EC on already differentiated epithelial cells in their natural context. We found that EC did not affect any of the epithelial markers we tested, including *Sftpc*, *Hopx*, *Foxj1*, *Scgb1a1* and *Muc5ac* (**Supplementary Figures 1A, B**). Collectively this indicates that EC affects the growth and differentiation of epithelial progenitors toward alveolar and airway phenotypes, but has little effect on the already differentiated epithelial cells themselves.

**Figure 2:**
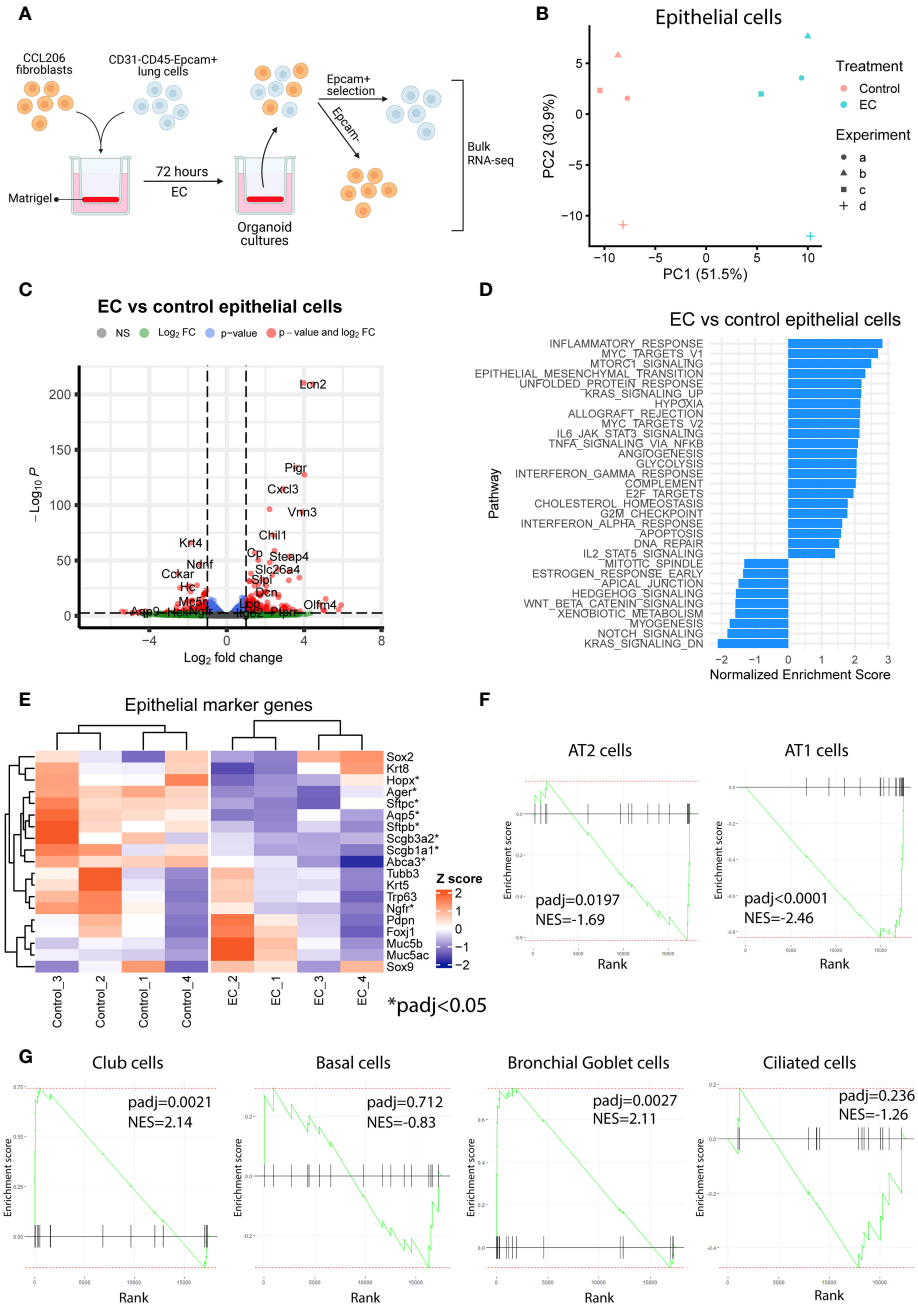
Exacerbation cocktail induces proliferation and epithelial differentiation defects already at early time point of organoid culture. **(A)** Mouse organoid cultures treated with exacerbation cocktail (EC) for 72 hours were re-sorted into Epcam+ (epithelial) cells and Epcam- cells (CCL206 fibroblasts), which were bulk RNA-sequenced separately (created using BioRender.com). **(B)** Principal component analysis (PCA) plot of epithelial cells originating from control or EC-treated mouse organoid cultures (N=4). **(C)** Volcano plot of genes altered by EC in epithelial cells from organoid cultures (cut-offs used: padj<0.05 and logFold change>1). **(D)** List of pathways significantly altered by EC in epithelial cells from mouse organoid cultures (positive enrichment score signals upregulation, negative enrichment score indicates downregulation). **(E)** Heatmap of epithelial marker genes in epithelial cells from organoid cultures exposed to vehicle control or EC. Individual genes marked with * are significantly altered by EC treatment (padj<0.05). **(F)** Enrichment plots of alveolar epithelial cell signatures from epithelial cells re-isolated from organoid cultures. Epithelial signature scores were determined using the marker genes associated with specific epithelial subtypes (from the human lung cell atlas (18)). Reported normalized enrichment scores (NES) and p adjusted values (padj) were obtained from pathway analysis using the epithelial cell type pathway definitions. **(G)** Enrichment plots of proximal epithelial cell signatures.

To explore the transcriptional effects of EC on organoids in more detail and to capture the early events associated with EC exposure, we exposed mouse organoid cultures (mouse Epcam+ cells co-cultured with CCL206 fibroblasts) to EC for 72 hours, after which we re-sorted the cells back into epithelial (Epcam+) and CCL206 fibroblast (Epcam-) fractions and subjected them to bulk RNA-sequencing separately (**Figure 2A**). The principal component analysis (PCA) plot indicated that epithelial cells grown in organoid cultures exposed to EC had a different gene signature compared to vehicle control (**Figure 2B**). Indeed, differential expression analysis revealed that EC induced 767 differentially expressed genes (DEGs) in epithelial cells (padj<0.05) (**Figure 2C**). Pathway analysis showed that pathways associated with inflammation (such as *inflammatory response, allograft rejection, IL-6/JAK/STAT3 signaling, TNFA signaling via NFKB*) and with proliferation (including *Myc targets, E2F targets* and *G2M checkpoint*) were significantly enriched in EC-exposed epithelial cells (**Figure 2D**). We note that *Epithelial mesenchymal transition* was upregulated, potentially related to the decrease in epithelial marker genes, and that pathways associated with lung development and repair including *Hedgehog signaling, Wnt/beta-catenin signaling* and *Notch signaling* (11) were reduced (**Figure 2D**). Interestingly, after 72 hour of EC-exposure, epithelial cells already had a reduced expression of various epithelial marker genes, including *Sftpc, Abca3, Aqp5, Ager* and *Scgb1a1* (**Figure 2E**). We also assessed changes in epithelial cell signatures, which were based on the marker genes associated with each epithelial subtype. These epithelial signatures were used to run pathway analysis, which revealed that the AT2 and AT1 cell signatures were significantly reduced, whereas the club cell and bronchial goblet cell signatures were enriched (**Figures 2F, G**). These results indicate that EC affects various cellular processes and pathways associated with repair including proliferation and differentiation already at an early time point.

### The epithelial progenitor support function of fibroblasts is altered by the exacerbation cocktail

**Figure 3:**
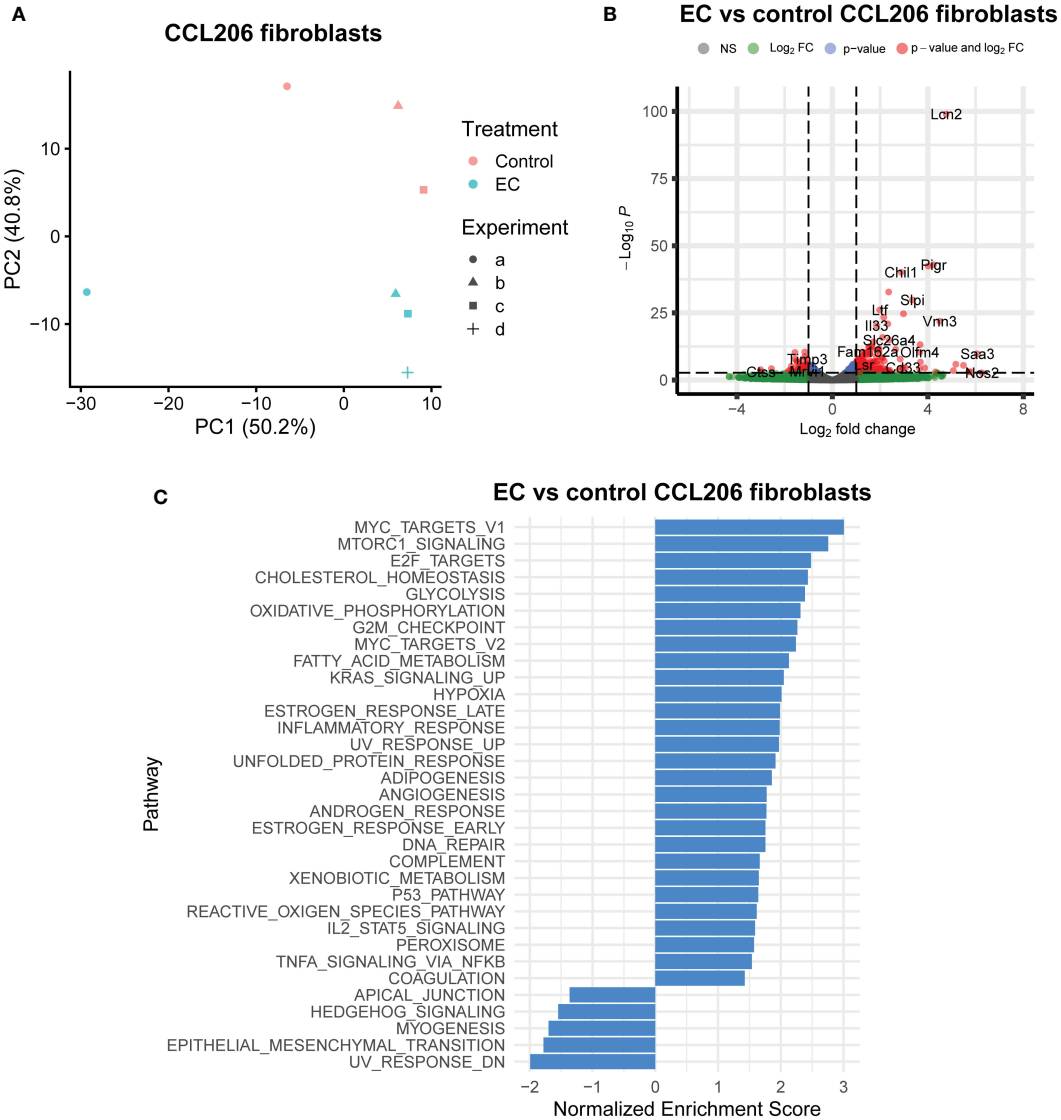
Exacerbation cocktail alters the expression of epithelial supportive factors in fibroblasts. Mouse organoid cultures were exposed to exacerbation cocktail (EC) for 72 hours, after which they were disrupted and re-sorted back into Epcam+ epithelial cells and CCL206 fibroblasts. Both cell fractions were bulk RNA-sequenced separately. **(A)** Principal component analysis (PCA) plot of CCL206 fibroblasts from mouse organoid cultures (for control: N=3 (one sample was excluded due to low quality RNA), for EC: N=4). **(B)** Volcano plot of genes altered by EC in CCL206 fibroblasts from organoid cultures (cut-offs used: padj<0.05 and logFold change>1). **(C)** Significantly altered pathways by EC in CCL206 fibroblasts from mouse organoid cultures (positive enrichment indicates upregulation, negative enrichment shows downregulation).

We also assessed the response of the fibroblasts from mouse organoid cultures exposed to EC for 72 hours. The PCA plot (**Figure 3A**) demonstrated that CCL206 fibroblasts also responded to EC treatment, and differential expression analysis showed that 533 genes were differentially expressed (padj<0.05) (**Figure 3B**). Pathway analysis showed that several pathways associated with proliferation (such as *MYC targets, E2F targets* and *G2M targets)* and metabolism *(cholesterol homeostasis, glycolysis, oxidative phosphorylation)* were upregulated. In addition, processes related to signaling pathways including *MTORC1 signaling, KRAS signaling, p53 pathway, IL2/STAT5 signaling* and *TNF-α signaling via NFKB* were enriched, whereas *Hedgehog signaling* was downregulated (**Figure 3C**). These data indicate that the cocktail not only affected epithelial cells, but also had a large effect on the fibroblasts in the organoid culture system (**Figures 3A–C**). Next, we pre-treated CCL206 fibroblasts with EC for 24 hours, washed the cells, after which we co-cultured them with mouse Epcam+ epithelial cells to form organoids. The co-culture itself was not exposed to EC. The fibroblast pre-treatment with EC was sufficient to induce formation of a significantly higher number of organoids (**Supplementary Figures 2A, B**), though organoid size was not affected (**Supplementary Figure 2C**). These data indicate a role for the fibroblasts in governing, at least part of, the EC effect on the epithelial cells.

**Figure 4:**
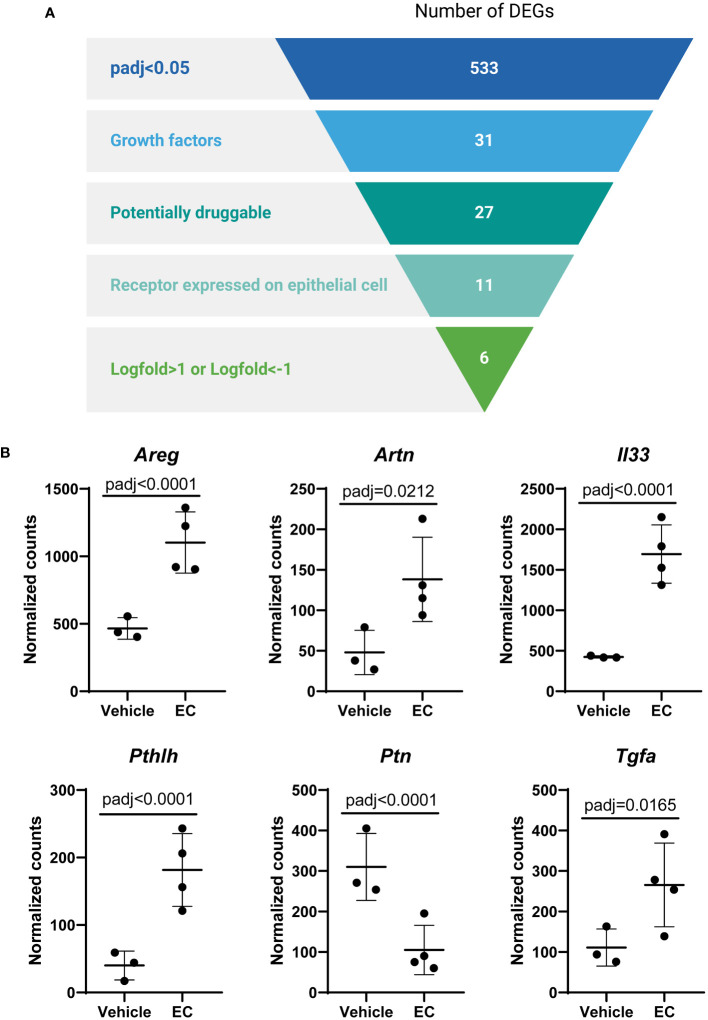
The expression of the growth factors amphiregulin, IL-33 and TGF-α is altered by the exacerbation cocktail in fibroblasts. **(A)** Target identification strategy. The list of differentially expressed genes (DEGs) from CCL206 fibroblasts originating from EC-exposed mouse organoids was used to identify growth factors produced by fibroblasts that potentially affect epithelial repair (created using BioRender.com). **(B)** Normalized raw counts extracted during DESeq2 analysis of the six genes that emerged from the filtering strategy in CCL206 fibroblasts from organoids (72 hour time point). P adjusted values obtained from DESeq2 analysis are shown.

In view of these data, we then aimed to identify specific mediators secreted by fibroblasts that were altered by EC and may impact epithelial progenitors. All genes that were significantly altered in the CCL206 fibroblast population re-isolated from EC-exposed organoid cultures were used as the starting point (using padj<0.05 as cut-off value). From these DEGs we extracted the growth factors, which were then selected for being potentially druggable and for having their corresponding receptor expressed on the Epcam+ epithelial cells. Finally, we selected the genes that had a logFold change of >1 or <-1, in order to identify the genes that were most highly changed (**Figure 4A**). Six genes emerged from this filtering process; *Areg, Artn, Il33, Pthlh, Ptn* and *Tgfa* (**Figure 4B**). As amphiregulin *(Areg)*, IL-33 *(Il33)* and TGF-α *(Tgfa)* are known to influence epithelial cell behavior (19–23), we selected these factors as potential mediators of epithelial cell changes in response to EC. The expression of *AREG, IL33* and *TGFA* was also evaluated in primary human non-COPD fibroblasts in mono-culture after 24 hours of EC-exposure. None of the genes were altered, although *IL33* showed a trend toward increased expression (p=0.0857) (**Supplementary Figure 3**), suggesting that co-culture is required for the EC effect on these genes to be evident.

### EGFR signaling is needed for organoid formation and for the effects of EC on organoid size

**Figure 5:**
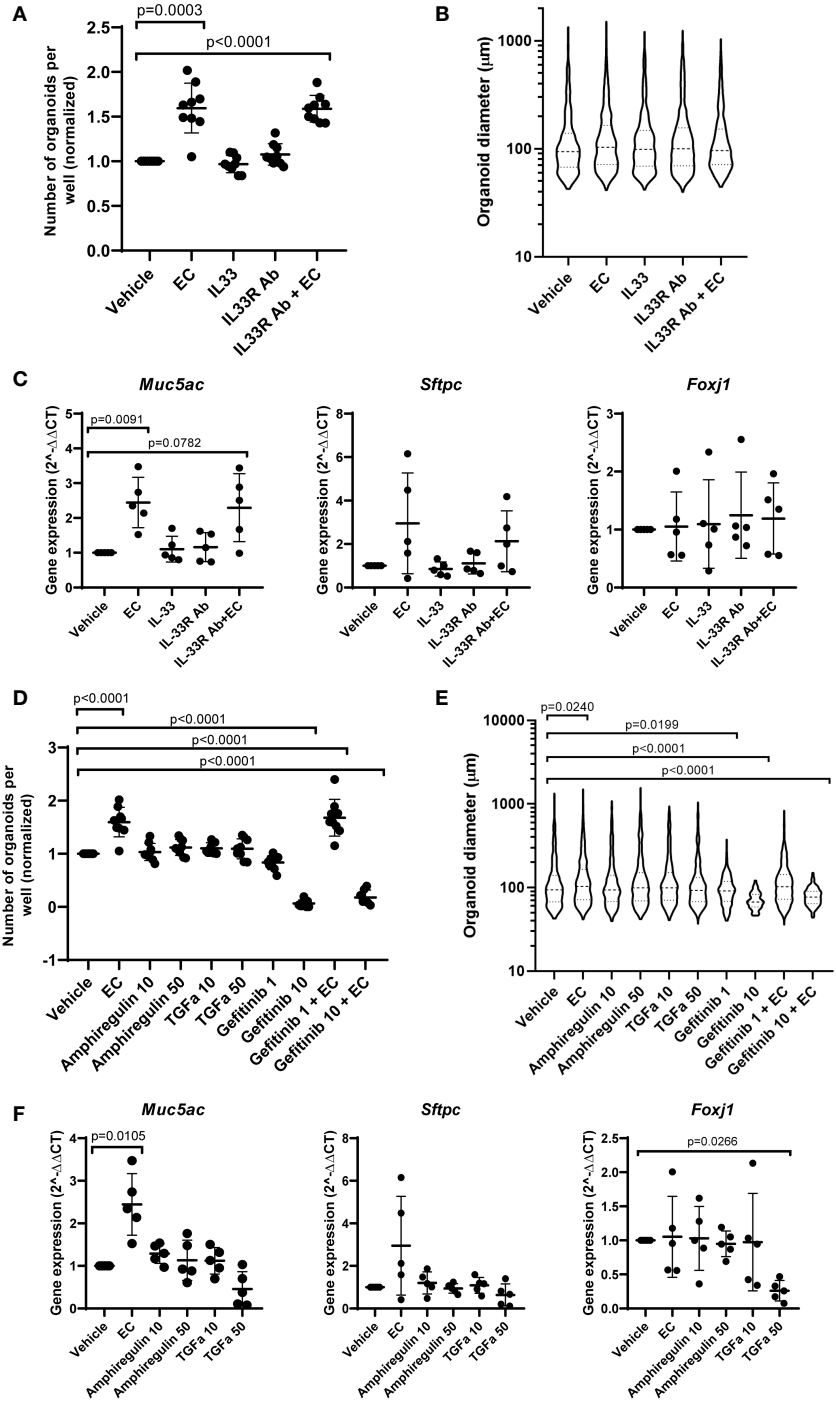
The effect of IL-33, amphiregulin and TGF-α on mouse organoids. **(A)** Number of mouse organoids formed on day 14 in response to EC, IL-33, IL-33 receptor antibody (IL33R Ab) or EC + IL-33R Ab (paired sample one way ANOVA on log transformed values, N=9). **(B)** Organoid size on day 14 in response to IL-33 signaling modulation (median is shown, Kruskal Wallis test, N=9). **(C)** The effect of IL-33 signaling modulation on the gene expression of epithelial marker genes *Muc5ac, Sftpc* and *Foxj1* in day 14 organoids (paired one way ANOVA on ΔCT values, N=5). **(D)** The number of organoids formed after 14 day stimulation with EC, 10 ng/ml or 50 ng/ml amphiregulin or TGF-α, or 1 or 10 μM gefitinib (one way ANOVA on log transformed values, N=7-9. Due to missing values, an unpaired statistical approach was used). **(E)** Organoid size measured on day 14 in response to amphiregulin, TGF-α or gefitinib exposure (median is shown, Kruskal Wallis test, N=7-9). **(F)** The effect of amphiregulin and TGF-α on the expression of epithelial marker genes *Muc5ac, Sftpc* and *Foxj1* (paired one way ANOVA on ΔCT values, N=5). For all data in this figure: the vehicle and EC values are the same values across the IL-33 and amphiregulin/TGF-α graphs. Additionally, the same samples of vehicle and EC were used for qPCR in **Figures 1**, **5**.

To assess the role of IL-33, amphiregulin and TGF-α in epithelial cell changes in response to EC, we treated mouse organoids with these factors and studied their response. We also used an antibody against ST2 (receptor for IL-33), and gefitinib, an inhibitor of epidermal growth factor receptor signaling (EGFR, receptor for amphiregulin and TGF-α). The activity of amphiregulin and TGF-α was confirmed by assessing the activation of downstream signaling pathways in CCL206 fibroblasts (data not shown), whereas the activity of IL-33 was previously validated (24). We found that neither IL-33 nor the antibody against the IL-33 receptor affected organoid number or size after 14 days of treatment (**Figures 5A, B**). EC increased the number of organoids formed (p=0.0003), and EC was still able to increase the number of organoids in the presence of the IL-33 receptor antibody (p<0.0001). The expression of epithelial marker genes *Aqp5, Hopx, Sftpc, Foxj1, Scgb1a1* and *Muc5ac* measured in day 14 organoids was not altered by either IL-33 or the antibody against the IL-33 receptor (**Figure 5C** and **Supplementary Figure 4A**). Whereas EC upregulated the expression of *Muc5ac* (p=0.0091), the combination of EC and IL-33 receptor antibody did not lead to a significant induction of *Muc5ac* expression, though there was a trend toward increase (p=0.0782).

After 14 days of exposure to amphiregulin or TGF-α, we observed that organoid formation was not affected (**Figure 5D**). We also found that the increased number of organoids formed in the presence of EC (p<0.0001) could not be inhibited by gefitinib at a concentration of 1 μM. However, gefitinib 10 μM almost completely abolished organoid formation (p<0.0001). The co-stimulation of EC and gefitinib 10 μM also led to a significant reduction in the number of organoids (p<0.0001). A similar trend was observed in organoid size (**Figure 5E**). Amphiregulin and TGF-α both did not have an effect, but gefitinib 1 μM and 10 μM both reduced organoid size (p=0.0199 and p<0.0001 respectively). Organoid size was increased by EC treatment (p=0.0240), which we showed in initial experiments as well. Interestingly, the co-stimulation of EC and gefitinib 10 µM resulted in a significantly decreased organoid size compared to vehicle control (p<0.0001). Finally, we evaluated the effect of amphiregulin and TGF-α on the expression of epithelial marker genes (**Figure 5F** and **Supplementary Figure 4B**). We note that organoids exposed to gefitinib could not be included in the gene expression studies because of low amounts of RNA isolated (likely due to the low number and small size of organoids formed in the presence of gefitinib). Whereas amphiregulin and TGF-α had no effect on the expression of *Hopx, Sftpc, Scgb1a1* and *Muc5ac*, TGF-α 50 ng/ml significantly downregulated *Foxj1* (p=0.0266) and increased the expression of *Aqp5* (p=0.0316).

## Discussion

The increased inflammatory response during an exacerbation in COPD is believed to have several deleterious effects including the worsening of airflow limitation (10), but whether epithelial repair responses are also affected is unknown. As epithelial progenitor cells are instructed by their micro-environment (11), we hypothesized that progenitors develop an altered response to the pro-inflammatory soluble factor milieu during exacerbations. We used EC, a combination of inflammatory mediators that are increased during a COPD exacerbation compared to stable COPD (IL-1β, IL-6, IL-8, TNF-α) to mimic the soluble factor microenvironment during an exacerbation. We showed that whereas regeneration initiation and proliferation were stimulated by EC, epithelial differentiation was altered. We also found that the epithelial progenitor supportive function of fibroblasts was altered, possibly contributing to the observed effects on epithelial regeneration.

We first determined the number and size of the mouse organoids as a measure of epithelial progenitor cell activation and subsequent epithelial proliferation respectively. EC increased the number of organoids formed, indicating heightened activation of epithelial progenitor cells to initiate regeneration. EC exposure also led to larger organoids, suggesting epithelial proliferation following organoid formation was elevated as well. Though these were unexpected findings in the context of COPD exacerbations, pro-inflammatory cytokines, including the individual components of the cocktail (IL-1β, IL-6, IL-8 and TNF-α) were previously suggested to stimulate epithelial proliferation and/or survival in injury models (25–29), whereas chronic inflammation is associated with airway remodeling and alterations in the composition of the epithelium (30). In the context of epithelial repair, the duration of exposure to inflammatory cytokines is critical. This is supported by studies by Choi et al. demonstrating that initially IL-1β promotes the differentiation of AT2 cells into AT1 cells, but prolonged IL-1β exposure impairs the maturation toward AT1 cells, leaving the cells in a AT2-AT1 transition state (26). Whereas the persistence of an AT2-AT1 transition state has not been described in COPD, preliminary results indicate that the expression of *KRT8*, which is associated with AT2-AT1 transition, is upregulated in epithelial cells from COPD lungs compared to controls (31). Interestingly, exposure to urban air pollution, a trigger for COPD exacerbations (2, 32), was also found to increase the number of KRT8+ cells in the mouse lung (33).

We also examined whether EC affects epithelial differentiation. In the PCLS model EC had no impact on expression of epithelial marker genes, indicating differentiated epithelial cells are not acutely affected by EC at the transcriptional level. We did find, however, that the differentiation of epithelial progenitors into mature epithelial cell types is altered, as indicated by our studies in the organoid model. We observed a reduction in pro-SPC staining in the organoid after 14 days of EC exposure. In addition, after 72 hours of EC exposure, epithelial cells grown in mouse organoid cultures were enriched for the club cell and bronchial goblet cell signatures, whereas the AT2 and AT1 cell signatures were significantly decreased. Additionally, they had a decreased expression of various epithelial genes, including *Sftpc, Abca3*, *Ager*, *Aqp5* and *Scgb1a1.* The downregulation of *Scgb1a1* and the enrichment of the club cell signature seem contrasting. However, whereas *Scgb1a1* is one possible club cell marker, the club cell signature is based on the differential gene expression of multiple marker genes associated with club cells, and their combined responses indicate an enrichment of the club cell signature. The epithelial gene expression pattern changed when the organoids had been grown in the presence of EC for 14 days, as *Lamp3*, *Muc5ac* and *Muc5b* were upregulated at the 14 day time point. The observed shift in AT2 cell markers is likely due to temporal regulation as epithelial growth and differentiation progress over the course of 14 days. Whereas AT2 cells are likely dysfunctional in COPD and are thought to have an impaired regenerative capacity (34), the alveolar epithelium has not been studied extensively in the context of COPD exacerbations. Exacerbations have been suggested to contribute to emphysema progression (35, 36), but emphysema severity has also been associated with risk of exacerbations (37), making it challenging to determine cause or consequence. The upregulation of mucin genes that we observed relates to epithelial changes in COPD and COPD exacerbations. The increased expression of *Muc5ac* and *Muc5b* is in accordance with the hyperplasia of goblet cells, the main producers of mucins, and mucus hyperproduction, which are both well-established processes in COPD (10, 38–40). Moreover, increased sputum production is one of the symptoms that can worsen during a COPD exacerbation (41), and increased MUC5AC protein concentrations have been measured in the sputum of exacerbating COPD patients (42). This indicates that the inflammatory micro-environment during COPD exacerbations could contribute to abnormalities observed in COPD such as mucus hyperproduction.

Next, we aimed to investigate the transcriptional effects of EC on organoids using bulk RNA sequencing. The enrichment of pathways related to proliferation in the Epcam+ epithelial cells from EC-exposed organoid cultures supports the increase in number and size of organoids formed after EC stimulation. Additionally, the enrichment of *IL-6/JAK/STAT3 signaling* and *TNFA signaling via NFKB* indicate that epithelial cells are directly impacted by these components of EC. Pathways related to lung development and repair, including *Hedgehog signaling, Wnt/beta-catenin signaling* and *Notch signaling* were downregulated. These pathways all regulate epithelial differentiation, where hedgehog signaling stimulates ciliated cell differentiation and the expression of *Muc5ac* and *Muc5b* (43, 44), Wnt signaling induces AT2 cell differentiation and favors the formation of ciliated cells over club cells (45, 46), and Notch signaling increases the differentiation of club cells at the expense of ciliated cells (47). In addition, the inhibition of Notch signaling is associated with the differentiation of alveolar cell types (48–50). Thus, the downregulation of all three of these pathways is likely related to the altered expression of various epithelial marker genes including *Sftpc, Aqp5* and *Scgb1a1* in epithelial cells from organoid cultures after 72 hour EC exposure. Moreover, there is evidence of hedgehog, Wnt and Notch signaling dysregulation in COPD (43, 51–56).

The regenerative response of epithelial progenitor cells is known to be coordinated by supportive cell types including fibroblasts, which can influence epithelial progenitor cells through paracrine signaling using factors such as FGFs and HGF (11). Therefore, we evaluated whether the observed effects of EC on organoids was mediated by an altered fibroblast response., Indeed, we show that the pre-exposure of CCL206 fibroblasts to EC is enough to increase the number of organoids formed, providing support that EC acts partially by altering the support function of fibroblasts. This is in accordance with previous studies where IL-1β, one of the components of EC, was suggested to exert its effects on organoids at least partially through modulating the supportive stromal cells (26, 27).

Finally, we aimed to identify specific mediators secreted by fibroblasts that were altered by EC and may impact epithelial progenitors. Using our filtering strategy we selected IL-33, amphiregulin and TGF-α as possible mediators of the EC effect and assessed their impact on epithelial regeneration. We demonstrated that IL-33 signaling did not impact organoid formation, size or differentiation. This is in apparent contrast with previous studies reporting that IL-33 is beneficial for epithelial repair. However, IL-33 was shown to regulate epithelial repair indirectly, for example by stimulating the release of epithelial growth factors by macrophages and regulatory T cells (21). Our results indicate that IL-33 may not impact epithelial cells directly. We also found that organoid formation and size was not affected by amphiregulin and TGF-α, which is in contrast with previous studies showing the mitogenic effect of amphiregulin and TGF-α on lung epithelial cells (22, 23, 57). Importantly, it was shown that amphiregulin was not needed for epithelial proliferation after naphthalene-induced lung injury (58), and the impact of amphiregulin on the proliferation of primary embryonic lung epithelial cells was found to be dependent on the presence of mesenchymal cells and the type of ECM that was present (59). This indicates that the modeling system used is critical for the mitogenic effect of amphiregulin. We also inhibited the receptor for amphiregulin and TGF-α (EGFR) using gefitinib. Gefitinib reduced organoid size, and gefitinib 10 uM was able to block the increase in organoid size induced by EC. This suggests that EGFR signaling is required for the enlargement of the organoids by EC. Moreover, gefitinib 10 uM almost completely blocked organoid formation, highlighting the importance of EGFR signaling for organoid initiation. Finally, we demonstrated that TGF-α modulated the expression of epithelial marker genes *Foxj1* and *Aqp5*. As TGF-α impacted different epithelial cell markers than EC, this suggests that TGF-α modulates signaling events within epithelial cells that are likely unrelated to the effects of EC.

While expanding knowledge using multicellular model systems it must be acknowledged that the current study had several limitations. Firstly, COPD exacerbations are recognized to be heterogeneous in terms of severity, triggering factor and inflammatory responses (60). Thus, EC is not an accurate representation of the soluble factor micro-environment in all exacerbations, but merely functions as a model of the general increased inflammatory response during an exacerbation. Our EC cocktail was also based on sputum concentrations and not on cytokine concentrations in the distal lung as such data are not available. Additionally, inflammatory cells including neutrophils, macrophages and CD8+ lymphocytes play an important role in COPD (30), but they were not included in the modeling systems used. The interactions between these inflammatory cells and EC in the context of lung epithelial repair would be interesting to unravel in future studies. Furthermore, our transcriptional analyses indicated that EC had a large effect on the gene expression of epithelial cells re-isolated from organoids, but further studies exploring the transcriptional impact of EC on specific epithelial subtypes are warranted. Finally, we studied the effect of EC on epithelial cells with a healthy background, whereas in COPD, both the cells and the micro-environment are changed even in the stable state. It would therefore be interesting to assess the effect of EC on diseased cells or on cells that were first exposed to a stimulus such as cigarette smoke extract or elastase as a COPD modeling system.

In conclusion, EC stimulates the activation of epithelial progenitors and subsequent epithelial proliferation, but it alters the dynamics of epithelial differentiation. The epithelial progenitor support function of fibroblasts is changed in the presence of EC, which may contribute to the altered formation, growth and differentiation of the organoids. The alterations in epithelial cell differentiation induced by the inflammatory microenvironment during a COPD exacerbation could contribute to abnormalities observed in COPD including goblet cell hyperplasia and mucus hypersecretion. In addition, our findings hold relevance to the understanding of the interplay between inflammatory cytokines and epithelial progenitor responses in development and repair.

## Data availability statement

The datasets presented in this study can be found in online repositories. The names of the repository/repositories and accession number(s) can be found below: https://www.ncbi.nlm.nih.gov/geo/, GSE245259.

## Ethics statement

The procedure for obtaining human lung tissue was in accordance to the Research Code of the University Medical Center Groningen (UMCG), as stated on https://umcgresearch.org/w/research-code-umcg as well as national ethical and professional guidelines Code of Conduct for Health Research (https://www.coreon.org/wp-content/uploads/2023/06/Code-of-Conduct-for-Health-Research-2022.pdf). The use of left-over lung tissue in this study was not subject to Medical Research Human Subjects Act in the Netherlands, as confirmed by a statement of the Medical Ethical Committee of the University Medical Center Groningen and therefore exempt from consent according to national laws (Dutch laws: Medical Treatment Agreement Act (WGBO) art 458/GDPR art 9/UAVG art 24). All donor material and clinical information were deidentified prior to experimental procedures, blinding any identifiable information to the investigators. Human lung tissue was obtained from excess lung tissue surplus to clinical care requirements derived from lung resection and lung transplant procedures. Histologically normal lung tissue from tumor resection surgery (non-COPD) was obtained from an area as far away as possible from the tumor and all tissues were examined by an experienced pathologist to exclude the presence of abnormalities. All animal procedures were approved by the local Animal Care and Use committee of the University of Groningen and performed according to the national guidelines under approval number AVD1050020209205.

## Author contributions

RK: Conceptualization, Data curation, Formal analysis, Investigation, Visualization, Writing – original draft. KG-K: Conceptualization, Supervision, Writing – review & editing. RF-M: Formal analysis, Data curation, Writing – review & editing.. RO: Investigation, Writing – review & editing. NA-A: Investigation, Writing – review & editing. JB: Conceptualization, Funding acquisition, Supervision, Writing – review & editing. RG: Conceptualization, Funding acquisition, Supervision, Writing – review & editing.
