# Supplementary material for: The disruptive effects of COPD exacerbation-associated factors on epithelial repair responses
Source: Front Immunol. 2024 Jun 7;15:1346491. doi: 10.3389/fimmu.2024.1346491 (PMC11193328; doi:10.3389/fimmu.2024.1346491)
Supplement: Supplementary file 1 [file DataSheet_1.docx]

**Supplementary tables**

Table S1: primer sequences used for gene expression studies on human fibroblasts

| **Gene** |  | **Sequence (5’-3’)** |
| --- | --- | --- |
| *B2M* | Fw | TGG AGG CTA TCC AGC GTA CT |
|  | Rv | CGG ATG GAT GAA ACC CAG ACA |
| *SDHA* | Fw | GCA TGC CAG GGA AGA CTA CA |
|  | Rv | ACG GGT CTA TAT TCC AGA GTG AC |
| *HMBS* | Fw | TGGACCTGGTTGTTCACTCCTT |
|  | Rv | CAACAGCATCATGAGGGTTTTC |
| *AREG* | Fw | GAG CCG ACT ATG ACT ACT CAG A |
|  | Rv | TCA CTT TCC GTC TTG TTT TGG G |
| *IL33* | Fw | TGA ATC AGG TGA CGG TGT TGA |
|  | Rv | AGC TCC ACA GAG TGT TCC TTG |
| *TGFA* | Fw | AGG TCC GAA AAC ACT GTG AGT |
|  | Rv | AGC AAG CGG TTC TTC CCT TC |

Table S2: primer sequences used for gene expression studies on mouse organoids and PCLS

| **Gene** |  | **Sequence (5’-3’)** |
| --- | --- | --- |
| *Rpl13a* | Fw | AGA AGC AGA TCT TGA GGT TAC GG |
|  | Rv | GTT CAC ACC AGG AGT CCG TT |
| *B2m* | Fw | ATG GGA AGC CGA ACA TAC TG |
|  | Rv | CAG TCT CAG TGG GGG TGA AT |
| *Actb* | Fw | ATC GTG CGT GAC ATC AAA GA |
|  | Rv | ATG CCA CAG GAT TCC ATA CC |
| *Hopx* | Fw | CGACTTTCAGTGGTTCCTGC |
|  | Rv | GTGTGGAAGTCTGGGCGAG |
| *Pdpn* | Fw | ACCGTGCCAGTGTTGTTCTG |
|  | Rv | AGCACCTGTGGTTGTTATTTTGT |
| *Aqp5* | Fw | CTC ACT GGG TCT TCT GGG TAG |
|  | Rv | TGC CGG TCA GTG TGC C |
| *Sftpc* | Fw | GGAGCACCGGAAACTCAGAA |
|  | Rv | GGAGCCGCTGGTAGTCATAC |
| *Lamp3* | Fw | TTG ACC CCA GCC TAA CCC AT |
|  | Rv | TCT TTC CCT GGT AAG TCT TCT CTG T |
| *Krt8* | Fw | GCCTGGTGGAGGACTTCAAGA |
|  | Rv | GCAACTCACGGATCTCCTCTTCAT |
| *Scgb1a1* | Fw | GGCCCTCCTCATGGAATCAG |
|  | Rv | GCATTTTGCAGGTCTGAGCC |
| *Muc5ac* | Fw | GAG ATG GAG GAT CTGG |
|  | Rv | GCA GAA GCA GGG AGT GGT AG |
| *Muc5b* | Fw | TCCCTAGCATGAGCGCCTTA |
|  | Rv | CCACGACGCAGTTGGATGTT |
| *Foxj1* | Fw | CGG CCA TCT ACA AGT GGA TCA |
|  | Rv | CTT GAA GGC CCC ACT GAG CA |
| *Tekt1* | Fw | ACA GTG CGA AGT GGT AGA CG |
|  | Rv | GCT GCG TGA TGG CAT TTT CA |

**Supplementary figures**





**Figure S1: Epithelial marker genes are not affected by exacerbation cocktail exposure in precision cut lung slices.** Mouse precision cut lung slices (PCLS) were incubated with exacerbation cocktail (EC) for 48 hours, after which gene expression studies were performed using qPCR on RNA extracted from the whole slice. Data are presented as 2^-ΔΔCT^ values, normalized to the vehicle control. (A) The expression of alveolar epithelial markers *Hopx* and *Sftpc* (paired samples Wilcoxon test on ΔCT values, N=10)*.* (B) Airway epithelial marker genes *Scgb1a1, Muc5ac* and *Foxj1* (paired samples Wilcoxon test on ΔCT values, N=10).


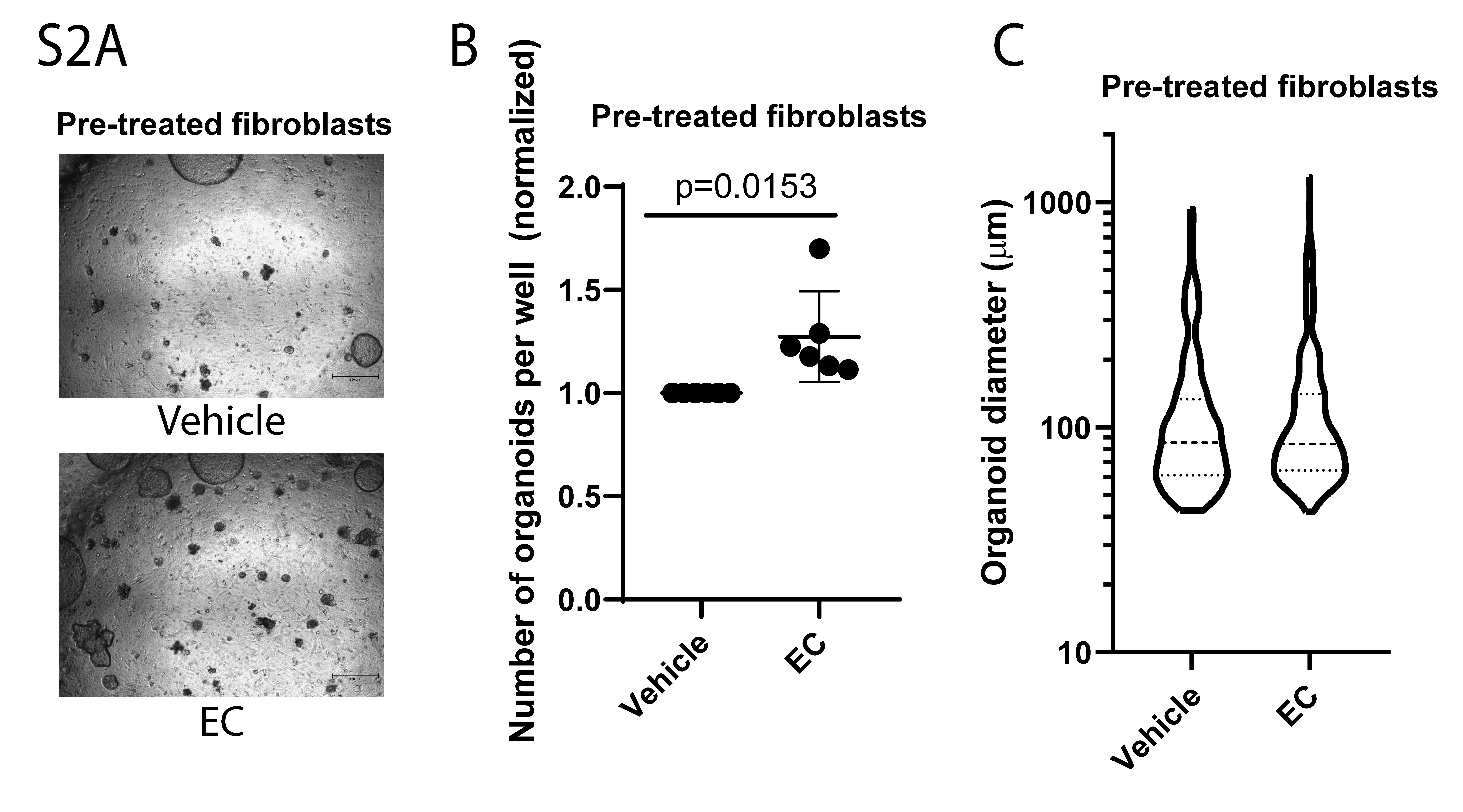


**Figure S2: Pre-exposure of CCL206 fibroblasts with exacerbation cocktail enhances organoid formation.** CCL206 fibroblasts were pre-treated with exacerbation cocktail (EC) for 24 hours and then mixed with freshly isolated mouse Epcam+ epithelial cells to form organoids. The organoids were not treated during the 14 days of culture. (A) Representative brightfield images of day 14 mouse organoids with pre-treated fibroblasts. Scale bar = 500 μm. (B) Number of mouse organoids formed on day 14, normalized to vehicle control pre-treatment (paired t-test on log-transformed data, N=6). (C) The effect of fibroblast pre-treatment with EC on organoid size (median is shown, Mann-Whitney test, N=6).


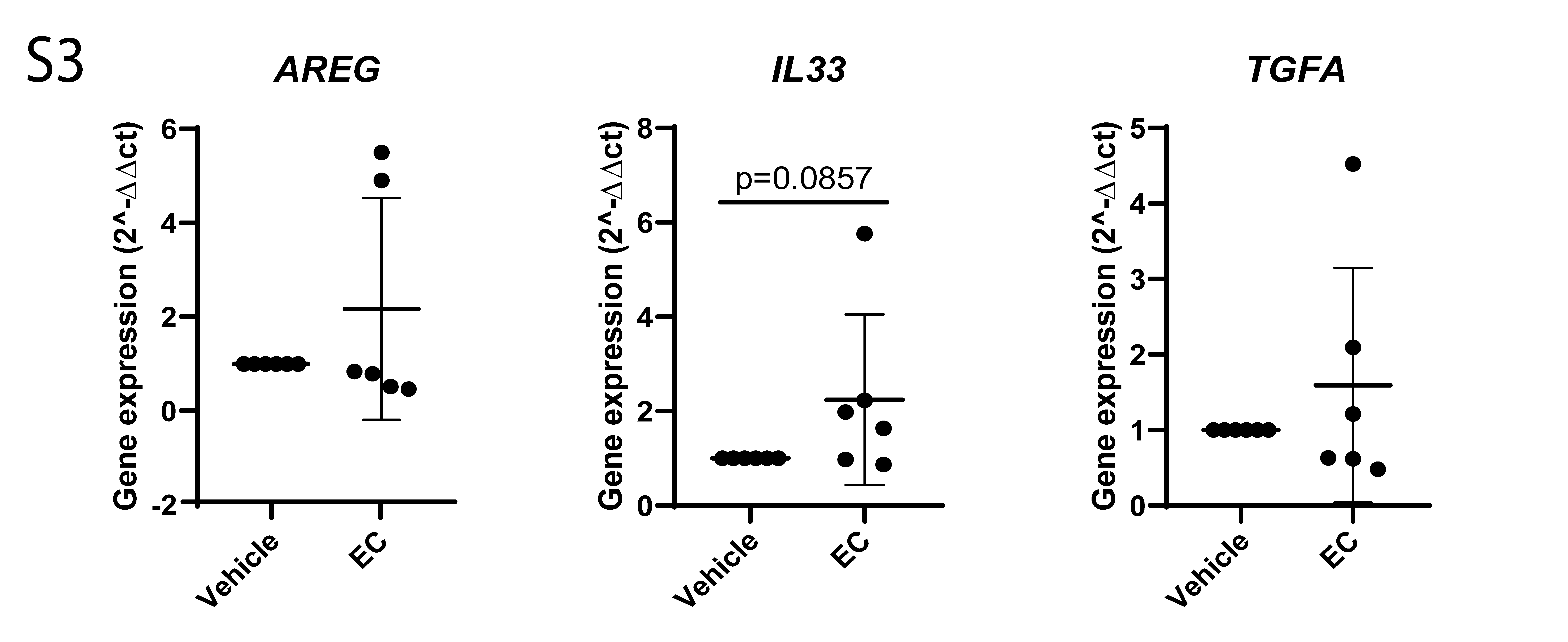


**Figure S3: The effect of exacerbation cocktail on the expression of amphiregulin, IL-33 and TGF-α in human fibroblasts.** Expression of the three selected genes *(AREG, IL33* and *TGFA)* in primary human non-COPD fibroblasts after 24 hour EC-exposure measured with qPCR. Data are presented as 2^-ΔΔCT^ values, normalized to vehicle control (paired t-test on ΔCT values, N=6).

**

**

**Figure S4: Epithelial marker gene expression in mouse organoids exposed to IL-33, amphiregulin and TGF-α.** Mouse organoids were cultured for 14 days after which RNA was isolated and epithelial marker expression was assessed using qPCR. (A) The expression of epithelial marker genes *Aqp5, Hopx* and *Scgb1a1* in day 14 organoids in response to IL-33 signaling modulation (for *Aqp5* and *Scgb1a1*: paired one way ANOVA on ΔCT values. For *Hopx:* paired Friedman test on ΔCT values. All: N=5). (B) The effect of 10 ng/ml or 50 ng/ml amphiregulin or TGF-α on the expression of *Aqp5, Hopx* and *Scgb1a1* in day 14 organoids (For *Aqp5* and *Scgb1a1:* paired one way ANOVA on ΔCT values. For *Hopx:* paired Friedman test on ΔCT values. All genes: N=5).
